# Reduction of Ischemia/Reperfusion Injury With Bendavia, a Mitochondria-Targeting Cytoprotective Peptide

**DOI:** 10.1161/JAHA.112.001644

**Published:** 2012-06-22

**Authors:** Robert A. Kloner, Sharon L. Hale, Wangde Dai, Robert C. Gorman, Takashi Shuto, Kevin J. Koomalsingh, Joseph H. Gorman, Ruben C. Sloan, Chad R. Frasier, Corinne A. Watson, Phillip A. Bostian, Alan P. Kypson, David A. Brown

**Affiliations:** Heart Institute of Good Samaritan Hospital, University of Southern California, Los Angeles (R.A.K., S.H., W.D.); Keck School of Medicine, Division of Cardiovascular Medicine, University of Southern California, Los Angeles (R.A.K., W.D.); Department of Surgery, University of Pennsylvania, Glenolden (R.C.G., T.S., K.J.K., J.H.G.); Department of Kinesiology, East Carolina University, Greenville, NC (R.C.S.); Department of Physiology, East Carolina University, Greenville, NC (C.R.F., D.A.B. C.A.W., P.A.B); Department of Cardiovascular Sciences, Brody School of Medicine, East Carolina University, Greenville, NC (A.P.K.)

**Keywords:** infarction, mitochondria, peptide, cardioprotection

## Abstract

**Background:**

Manifestations of reperfusion injury include myocyte death leading to infarction, contractile dysfunction, and vascular injury characterized by the “no-reflow” phenomenon. Mitochondria-produced reactive oxygen species are believed to be centrally involved in each of these aspects of reperfusion injury, although currently no therapies reduce reperfusion injury by targeting mitochondria specifically.

**Methods and Results:**

We investigated the cardioprotective effects of a mitochondria-targeted peptide, Bendavia (Stealth Peptides), across a spectrum of experimental cardiac ischemia/reperfusion models. Postischemic administration of Bendavia reduced infarct size in an in vivo sheep model by 15% (*P*=0.02) and in an ex vivo guinea pig model by 38% to 42% (*P*<0.05). In an in vivo rabbit model, the extent of coronary no-reflow was assessed with Thioflavin S staining and was significantly smaller in the Bendavia group for any given ischemic risk area than in the control group (*P*=0.0085). Myocardial uptake of Bendavia was ≈25% per minute, and uptake remained consistent throughout reperfusion. Postischemic recovery of cardiac hemodynamics was not influenced by Bendavia in any of the models studied. Isolated myocytes exposed to hypoxia/reoxygenation showed improved survival when treated with Bendavia. This protection appeared to be mediated by lowered reactive oxygen species–mediated cell death during reoxygenation, associated with sustainment of mitochondrial membrane potential in Bendavia-treated myocytes.

**Conclusions:**

Postischemic administration of Bendavia protected against reperfusion injury in several distinct models of injury. These data suggest that Bendavia is a mitochondria-targeted therapy that reduces reperfusion injury by maintaining mitochondrial energetics and suppressing cellular reactive oxygen species levels. **(*J Am Heart Assoc*. 2012;1:e001644 doi: 10.1161/JAHA.112.001644.)**

## Introduction

Early and successful myocardial reperfusion with primary percutaneous coronary intervention remains the most effective strategy for reducing the size of a myocardial infarct and improving clinical outcome. Reperfusion injury is a major issue in patients who receive percutaneous coronary intervention or thrombolysis or have spontaneous reperfusion for ST-segment myocardial infarction. Two major manifestations of reperfusion injury are myocyte death (infarction) and microvascular damage (no-reflow). Given that long-term prognosis has been linked to both size of infarction^1–3^ and the extent of reflow,^4,5^ strategies aimed to decrease infarct size or limit the amount of no-reflow have significant potential as adjuvant therapies.

Reactive oxygen species (ROS) are centrally involved in the development of infarction and no-reflow. Augmented production of ROS during early reperfusion contributes to both myocyte death^6,7^ and microvascular dysfunction leading to no-reflow.^8,9^ Elevated ROS increase the probability of opening of the mitochondrial permeability transition pore,^10^ which is followed by bioenergetic collapse and ultimately cell death. Given that mitochondrial ROS production is high in early reperfusion, therapies that directly (and effectively) target mitochondrial ROS production are ideal in this setting. A novel class of cell-permeable peptides has been developed that selectively target mitochondria. The Szeto-Schiller (SS) peptides are relatively small water-soluble molecules that contain a similar structural motif of alternating basic and aromatic residues, which allows them to freely cross cell membranes (despite a 3+ net charge at physiological pH).^11^ Studies with fluorescent and radiolabeled SS peptides indicate that they localize to mitochondria and concentrate at inner mitochondrial membranes.^12^ One particular peptide, Bendavia (an analogue of SS-02 and SS-31; Stealth Peptides), has been shown to reduce ROS levels in isolated mitochondria and to protect cultured cells against cell death induced by a variety of chemical stressors.^13^ Although these findings are promising, whether they relate to primary cardiac myocytes exposed to hypoxia/reoxygenation and translate to large animal models of ischemia/reperfusion (eg, infarction and no-reflow) is not known.

The purpose of the present study was to determine whether Bendavia could protect the myocardium from reperfusion injury. In a collaboration that spanned 3 independent laboratories, we used large animal models of ischemia/reperfusion, isolated perfused hearts, and myocyte hypoxia/reoxygenation experiments to determine if this mitochondria-targeting peptide attenuated cell death and no-reflow.

## Methods

The animals used in these studies were maintained in accordance with the Position of the American Heart Association on Research Animal Use (American Heart Association, 1985) and the Guide for Care and Use of Laboratory Animals (1996). Protocols in all 3 laboratories received prior approval by the institutional animal care and use committee of each individual institution (University of Pennsylvania, Good Samaritan Hospital, and East Carolina University). All sheep studies were performed by the Gorman Cardiovascular Research Group at the Glenolden Research Laboratory, Department of Surgery, University of Pennsylvania School of Medicine, Glenolden, PA. All rabbit studies were done in the Kloner Laboratory at the Good Samaritan Hospital, University of Southern California, Los Angeles. Studies using guinea pigs were done in the Brown Laboratory at the Brody School of Medicine, East Carolina University, Greenville, NC.

### Ischemia/Reperfusion Injury in Sheep Model

Male Dorsett hybrid sheep were randomly assigned to treatment with vehicle (n=8) or Bendavia (0.05 mg/kg per hour; n=11). The data are from 2 groups of male Dorsett hybrid sheep that were entered into a cyclosporine/Bendavia dose-assessment study. Data from the group of sheep that received the same dose of Bendavia as the rabbits (0.05 mg/kg per hour; n=11) and from all sheep treated with vehicle (n=8) are presented here. Sheep received a preanesthetic regimen of ketamine (10 mg/kg IV over 30 seconds), diazepam (1 mg/kg IV over 30 seconds), and glycopyrrolate (0.015 mg/kg IV bolus). Anesthesia was induced with inhaled isoflurane (1% to 3%). The sheep were pretreated with antiarrhythmic medication: lidocaine bolus followed by continuous intravenous infusion at ≈0.015 mg/min. A Swan-Ganz pulmonary artery catheter was inserted through a right internal jugular sheath and “floated” into the pulmonary artery to monitor pulmonary artery pressure, central venous pressure, and core temperature. A Millar catheter was inserted into the arterial sheath placed in the femoral artery to monitor left ventricular (LV) pressure. Body temperature was maintained between 38.8°C and 39.0°C in both groups throughout the protocol.

To create the myocardial infarction, snares were placed around the distal left anterior descending coronary artery and selected branches of the diagonals to achieve an ischemic risk zone of ≈20% to 25% of the LV. Occlusion of the coronary vessel(s) lasted 60 minutes and was followed by 180 minutes of reperfusion. Hemodynamic variables were recorded and analyzed at baseline, at the end of the ischemic period, and after 5 minutes and 180 minutes of reperfusion.

At 30 minutes of ischemia, intravenous infusions of either Bendavia or placebo were initiated and continued through ischemia and until the conclusion of the reperfusion phase. At the end of 3 hours of reperfusion, the coronary arteries were religated, and infarct size was assessed as previously described.^14^

### Statistical Analyses for Sheep Studies

Data summary and statistical analyses were performed with SAS (Version 9.3, Cary, NC). Differences between area at risk (AAR) and infarct size were assessed by the Student *t* test. Changes in hemodynamic variables over time were analyzed by repeated-measures analysis of variance (ANOVA). Analysis of covariance (ANCOVA) was used to test for a group effect on the regression model of necrotic zone and risk zone in sheep. Data are expressed as mean±SEM.

### No-Reflow and Infarct Size in Rabbit Model

The techniques used for the rabbit model of acute myocardial infarction in the Kloner laboratory have been described previously.^15^ Briefly, anesthetized, open-chest male New Zealand White rabbits (2.5 to 3.3 kg) were subjected to 30 minutes of coronary artery occlusion (CAO) followed by 3 hours of reperfusion. After 15 minutes of stabilization, baseline hemodynamic parameters and temperatures were obtained, with body temperatures maintained between 37.9°C and 38.1°C throughout all experiments. Rabbits were randomized to one of the following 4 groups:

**Group 1:** treatment at 20 minutes before reperfusion, 0.05 mg/kg per hour of Bendavia, intravenous infusion starting at 10 minutes after CAO and continuing throughout reperfusion
**Group 2:** treatment at 10 minutes before reperfusion, starting 20 minutes after CAO, 0.075 mg/kg per hour of Bendavia, intravenous infusion for the first 20 minutes, then 0.05 mg/kg per hour throughout reperfusion
**Group 3:** treatment starting immediately before coronary artery reperfusion (1 minute), 0.10 mg/kg per hour intravenous infusion for the first 20 minutes, then changed to 0.05 mg/kg per hour of Bendavia throughout reperfusion
**Group 4:** saline, an equivalent volume relative to Bendavia starting at 10 minutes after CAO and continuing throughout reperfusion.

The total volume infused was <6 mL.

The extent of the no-reflow zone was assessed by staining with Thioflavin S (Sigma-Aldrich), a fluorescent yellow dye that stains endothelium and serves as a marker of regional perfusion. Under ultraviolet light, the no-reflow zone appears as a nonfluorescent, dark area, and regions of perfusion appear brightly fluorescent. The risk zone was delineated by Unisperse blue pigment (Ciba-Geigy) and necrosis by triphenyltetrazolium chloride. Measurements of risk zone, no-reflow zone, and infarct size were calculated as previously described.^15^

Sixty-six rabbits entered the protocol with 64 successful studies: Group 1, n=15; Group 2, n=17; Group 3, n=17; and Group 4, n=15. Data from 2 hearts were excluded on the basis of the prospective exclusion criterion of ischemic risk zone size (AAR <10% of the LV).

### Statistical Analyses for Rabbit Study

Data from the 4 groups were analyzed. In addition, to assess the overall effects of treatment with Bendavia when administered after the onset of CAO but before reperfusion, infarct size and no-reflow data from the 3 treated groups (n=49) were combined and compared with that from the control group (n=15). A post hoc analysis was performed with a risk zone exclusion of <20% of the LV to study a population comparable to the sheep data.

Data summary and statistical analyses were performed with SAS (Version 9.3, Cary, NC). LV weight, infarct size, AAR, and area of no-reflow were compared by ANOVA. Changes in hemodynamic variables were analyzed by repeated-measures ANOVA. ANCOVA was used to test for a group effect on the regression model of no-reflow zone with risk zone. Infarct size and no-reflow data from the combined Bendavia-treated hearts versus control hearts were analyzed by *t* test and by ANCOVA. Data are expressed as mean±SEM.

### Ischemia/Reperfusion Injury in Guinea Pigs

Adult male guinea pigs (200 to 300 g) were anesthetized with a ketamine/xylazine cocktail (85/15 mg/kg, respectively; intraperitoneal delivery). Hearts were excised, placed on a modified Langendorff apparatus, and instrumented for the observation of electromechanical function, as previously described.^16,17^ Hearts were divided into the following treatment groups: (1) control followed by global ischemia/reperfusion (n=14); (2) administration of 1 nmol/L Bendavia in the perfusate beginning 10 minutes before index ischemia and for the entire reperfusion (n=9); (3) postischemic administration of 1 nmol/L Bendavia for the duration of reperfusion (n=9); and (4) postischemic administration of 0.2 μmol/L cyclosporine A (n=8). For groups 2 through 4, experimental compounds were dissolved in Krebs Henseleit buffer (Sigma-Aldrich) and placed in dedicated buffer reservoirs (gassed with 95%/5% O_2_/CO_2_, 37°C). Hearts were exposed to global no-flow ischemia by stopping perfusion for 20 minutes. At the end of the 120-minute reperfusion period, infarct size and arrhythmia scores were assessed as previously described.^16,17^

### Myocardial Uptake of Bendavia

A separate group of guinea pigs (n=7) was used to determine the extent of Bendavia uptake by the myocardium at baseline and throughout reperfusion. Hearts were perfused constantly with 1 nmol/L Bendavia as described above, and coronary effluent was collected through a pulmonary artery cannula. Samples were collected at baseline (before 20 minutes of global no-flow ischemia) and every 15 minutes for the first hour of reperfusion. At each time point, 2 samples of perfusate were obtained (1 mL of each): one from the buffer before entry into the perfusion cannula (input) and the other from the pulmonary cannula (output). Samples were immediately mixed with 100 μL of 5% formic acid and snap-frozen in liquid nitrogen, and Bendavia concentration was subsequently determined with liquid chromatography–mass spectrometry (Sciex API4000 LC-MS/MS system coupled with Shimadzu High-Performance Liquid Chromatography; Medpace Bioanalytical Laboratories, Cincinnati, OH). The calibration range of the assay was 0.1 to 100 ng/mL. Three levels of quality-control samples at low (0.3 ng/mL), mid (5 ng/mL), and high (80 ng/mL) concentrations were included in each batch of analysis. The chromatographic separations were achieved on a UniverSil 5 μm C18, 100 × 2.1 mm high-performance liquid chromatography column and a mobile phase gradient. The mass spectrometer was operated in positive electrospray ionization mode. The multiple reaction monitoring transition ions were *m/z* 640.3/303.3 for Bendavia. Peak-area integration was performed with Analyst software (version 1.5, Applied Biosystems).

The measured Bendavia concentration (in nanograms per milliliter) of each sample was converted to nanomolar (nmol/L) concentration by using molar weight of the free peptide (639.8 g/mol). Organ uptake (%) was calculated by using the standard formulation:


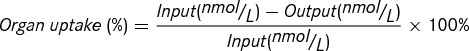


where Input is the Bendavia concentration in the perfusate buffer and Output is the Bendavia concentration in the pulmonary artery (coronary effluent). Uptake data are expressed as the percentage of Input Bendavia retained by the organ during single-pass perfusion.

### Myocyte Hypoxia/Reoxygenation Experiments

Guinea pig LV myocytes were isolated enzymatically from a total of 17 animals according to established protocols.^18^ Isolated primary cardiomyocytes were enzymatically isolated and incubated (95% O_2_ balance room air, 37°C) for 2 to 8 hours after dispersion. For cellular hypoxia/reoxygenation studies, isolated myocytes were placed in a perfusion chamber housed on the stage of a fluorescence microscope (Leica) for the study of cell survival and ROS production during cellular hypoxia/reoxygenation. The small-volume perfusion chamber (250 μL) used in the study is enclosed in glass to minimize gas exchange with the environment and is equipped with pacing electrodes for field stimulation (Warner Instruments). The chamber is housed on the microscope stage by a magnetic holder with heating filaments, allowing us to conduct our measurements at 37°C, and is connected to an in-line solution heater that delivers the superfusate via laboratory tubing that has very low oxygen permeability (Tygon F-4040-A).

For each myocyte experiment, cells were allowed to settle on the glass coverslips for ≥20 minutes before the initiation of perfusion. Differential interference contrast images of the myocytes were obtained at the beginning and end of each experiment. Cells were perfused with Tyrode's solution containing (in mmol/L): 140 NaCl, 10 HEPES, 5 KCl, 1 MgCl_2_, 1.8 CaCl_2_, and 10 glucose (pH=7.4, 37°C), delivered at a rate of 1.0 to 1.2 mL/min. Only rod-shaped myocytes that were responsive to field stimulation (an assessment of cell viability) were selected for hypoxia/reoxygenation studies. Myocytes were paced (4-ms duration, 1-Hz frequency, 10-V amplitude) for the duration of the hypoxia/reoxygenation protocol on the basis of observations that guinea pig myocardium maintains electrical activity through 20 minutes of ischemia.^19^

For the drug treatment groups, myocytes either were incubated for 10 minutes with 1 nmol/L Bendavia before being placed in the perfusion chamber (Bendavia group) or received no drug treatment (Control group). Myocytes received 5 minutes of baseline superfusion, after which the solution was switched to Tyrode's solution gassed with 100% argon (hypoxia solution) for 20 minutes. After 20 minutes of perfusion with hypoxia solution, the superfusate was switched back to normoxic Tyrode's to initiate reoxygenation. Cells were reoxygenated for 30 minutes or until cell death, whichever came first. When appropriate, the time of myocyte death during hypoxia/reoxygenation was noted by complete transition from rod-shaped to rounded, necrotic cell morphology.

Subsets of myocytes that underwent hypoxia/reoxy-genation were loaded with one of 2 fluorescent probes. Cells either received the ROS probe 5-(6)-chloromethyl-2,7-dichlorohydrofluorescein diacetate (CM-DCF, Invitrogen), or the ΔΨ_m_ sensor tetramethylrhodamine methyl ester (TMRM, Sigma) during the protocol. Another cohort of cells was exposed to hypoxia/reoxygenation with no fluorescent probe to determine if the fluorophores themselves influenced cell survival. Because the different flouorophores did not influence the proclivity to cell death during the protocol (when compared to unloaded cells), the data were pooled for the survival plot to include all cells exposed to hypoxia/reoxygenation.

### Cellular Hypoxia/Reoxygenation and Simultaneous ROS Production

Ventricular cardiomyocytes were loaded with 500 nmol/L of the fluorescent ROS probe CM-DCF (Invitrogen) for 10 minutes in the incubator before imaging. A subset of myocytes was incubated for 10 minutes with 1 nmol/L Bendavia (in addition to CM-DCF), also in the incubator. CM-DCF fluorescence was evoked by using light from a metal-halide lamp filtered to an excitation wavelength of 472 nm (band-pass filter width 30 nm), and emission was collected at 520 nm (band-pass filter width 36 nm). Emitted light was captured with a charge-coupled device camera, and images were acquired on a personal computer. To avoid photobleaching of the probe, sampling rate was set at 30-second intervals for the duration of the protocol. Our preliminary data indicated that this sampling rate and fluorophore concentration led to stable recordings in normoxic (nonstressed) myocytes for up to 50 minutes (data not shown).

For the CM-DCF analysis, changes in fluorescence intensity were quantified for each time point by subtracting the cell fluorescence (obtained via a region of interest drawn around the cell perimeter) from background fluorescence (obtained via a region of interest in an area adjacent to each myocyte). To account for unequal fluorophore loading across cells, each cellular fluorescence trace was normalized to baseline (prehypoxia) fluorescence intensity (F_o_) for each cell. Cell survival was monitored on the computer screen as the ROS signal was captured.

### Mitochondrial Membrane Potential (ΔΨ_m_) During Hypoxia/Reoxygenation

Separate experiments were conducted to determine the influence of Bendavia on ΔΨ_m_ during cellular hypoxia/reoxygenation. Isolated guinea pig ventricular myocytes were loaded with the ΔΨ_m_ sensor TMRM for 10 minutes before imaging. We used a TMRM concentration of 25 nmol/L, which is “nonquench” mode in this application. TMRM fluorescence was evoked by using an excitation wavelength of 560 nm (band-pass filter width 40 nm), and emission was collected at 645 nm (band-pass filter width 72 nm). TMRM fluorescence intensity was captured every 60 seconds throughout hypoxia and reoxygenation or until cell death, whichever came first.

### Statistical Analyses for Guinea Pig Studies

The myocyte survival curve was analyzed with a log-rank (Mantel-Cox) test. Infarct size and Bendavia uptake data for the guinea pig hearts were analyzed by ANOVA, followed by Newman-Keuls post hoc tests for between-group comparisons. For all comparisons, significance was noted if *P*<0.05.

## Results

### Effect of Bendavia on Ischemia/Reperfusion Injury Across Models

**Figure 1.**
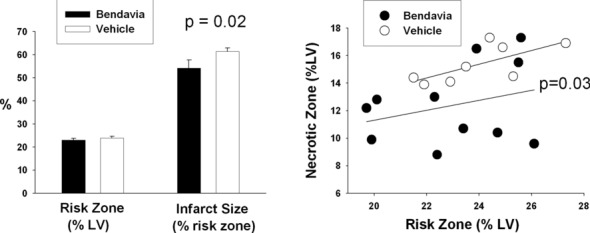
Left, Ischemic risk zone (% LV) and infarct size (% risk zone) in sheep exposed to in vivo ischemia/reperfusion (60 min/3 h). Bendavia (0.05 mg/kg per hour; black bars) was administered intravenously beginning 30 minutes before reperfusion (mean±SEM; *P*=0.02, *t* test). Right, Relationship between the extent of the ischemic risk zone (% LV) and the extent of necrosis (% LV) (*P*=0.03, ANCOVA).

The effect of Bendavia on infarct size in sheep is presented in Figure 1 left. The ischemic AAR was similar in both groups, comprising 24.0±0.7% of the LV in the vehicle group and 23.1±0.7% in the Bendavia-treated group. Treatment with Bendavia significantly reduced infarct size by 15.4%, from 64.1±1.5% of the risk zone in the vehicle group to 54.2±3.5% in Bendavia-treated sheep (*P*=0.02). Overall, for any given risk zone size, the extent of necrosis was lower in Bendavia-treated hearts (*P*=0.03; Figure 1 right).

**Figure 2.**
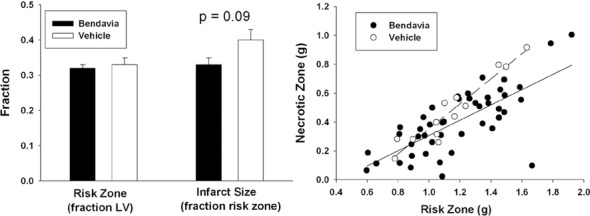
Left, Ischemic risk zone (fraction LV) and infarct size (fraction risk zone) in rabbits with risk zones >20% of the LV exposed to in vivo ischemia/reperfusion (30 min/3 h) in combined Bendavia treatment group and vehicle group (mean±SEM; *P*=0.09, *t* test). Note similarity to data observed in the sheep model (text, Figure 1). Right, Relationship between the extent of the ischemic risk zone (g) and the extent of necrosis (g) in combined Bendavia group and vehicle group. Regression lines: vehicle is indicated by dashed line; Bendavia, solid line.

In the rabbit model, the size of ischemic risk zone, expressed as a fraction of the LV, was similar in all groups (average of 0.30 to 0.32; *P*=NS). There were no statistically significant differences in mean values among the 4 groups in infarct size expressed as a fraction of the risk zone (Table 1). To assess the overall effects of treatment with Bendavia when administered after the onset of CAO but before reperfusion, infarct size from the 3 treated groups was combined and compared with that from the control group. Overall, there was a nonsignificant 11% reduction in infarct size with Bendavia treatment. A post hoc analysis was performed to examine infarct size in rabbits with larger infarcts (>20% of the LV; Table 2 and Figure 2) to parallel the sheep model, in which the risk regions were generally ≥20% of the LV. Infarct size in Bendavia-treated animals was reduced by 17.5%, from 0.40±0.03 of the ischemic risk zone to 0.33±0.02, resulting in a strong statistical trend (*P*=0.09) and consistent with the degree of myocardial protection demonstrated with sheep (Figure 1). Interestingly, Bendavia-induced reductions in infarct size appeared to be more prominent in hearts with larger at-risk zones, consistent with previous studies indicating that cardioprotective strategies may be most effective when the zone at risk is larger.^20,21^

**Table 1.** Risk Zone and Infarct Size in the Rabbit Model: 4 Treatment Groups

	Group 1 (n=15)	Group 2 (n=17)	Group 3 (n=17)	Group 4 (n=15)	*P*
Risk zone	0.30±0.02	0.32±0.02	0.31±0.02	0.30±0.02	0.89

Infarct size	0.33±0.03	0.32±0.04	0.31±0.04	0.36±0.04	0.74

Risk zone is given as fraction of LV; infarct size, fraction of risk zone.

Treatment groups: Group 1 received Bendavia starting at 20 minutes before coronary artery reperfusion; Group 2 received Bendavia starting at 10 minutes before reperfusion; Group 3 received Bendavia starting 1 minute before reperfusion; and Group 4 received saline. See text for details.

**Table 2.** Risk Zone and Infarct Size in Combined Treatment Group Versus Vehicle

	Bendavia	Vehicle	*P*
Risk zone ≥10% of LV	n=49	n=15	

Risk zone	0.31±0.01	0.30±0.02	0.65

Infarct size	0.32±0.02	0.36±0.04	0.30

Risk zone ≥20% of LV	n=46	n=13	

Risk zone	0.32±0.01	0.33±0.02	0.84

Infarct size	0.33±0.02	0.40±0.03	0.09

Risk zone is given as fraction of LV; infarct size, fraction of risk zone.

**Figure 3.**
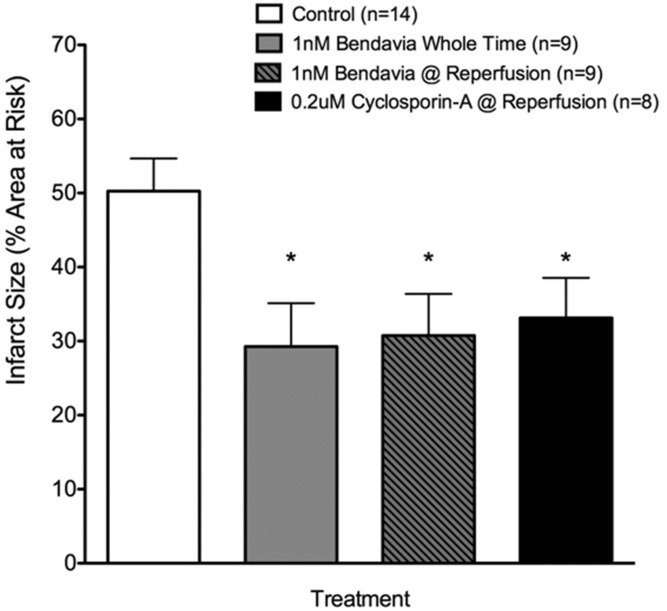
Infarct size in isolated guinea pig hearts exposed to global ischemia/reperfusion (20 min/2 h). Experimental compounds were either administered both before and after ischemia (whole time) or administered only at the onset of reperfusion (@ reperfusion) (mean±SEM; *P*<0.05 vs control, ANOVA followed by Newman-Keuls post hoc tests for between-group comparisons).

The effect of Bendavia on infarct size in guinea pigs is presented in Figure 3. In this model, the entire LV is hypoxic, so the risk area consists of the entire LV mass. After the ischemia/reperfusion protocol, untreated (control) guinea pighearts had infarct sizes of 50±4% of the AAR (n=14, Figure 3). Administration of 1 nmol/L Bendavia, either before ischemia (n=9) or at the onset of reperfusion only (n=9), significantly reduced infarct size to 29±6% and 31±6% AAR, respectively (*P*<0.05 versus control, ANOVA). Treatment with 0.2 μmol/L cyclosporine A at the onset of reperfusion also significantly reduced infarct size to 33±5% of the AAR (*P*<0.05 versus control, ANOVA; n=8). There were no differences in infarct size among treatment groups. Moreover, reductions in infarct size were more prominent, with larger at-risk zones in guinea pigs than in sheep and rabbits.^20,21^

### Effect of Bendavia on Coronary No-Reflow

**Figure 4.**
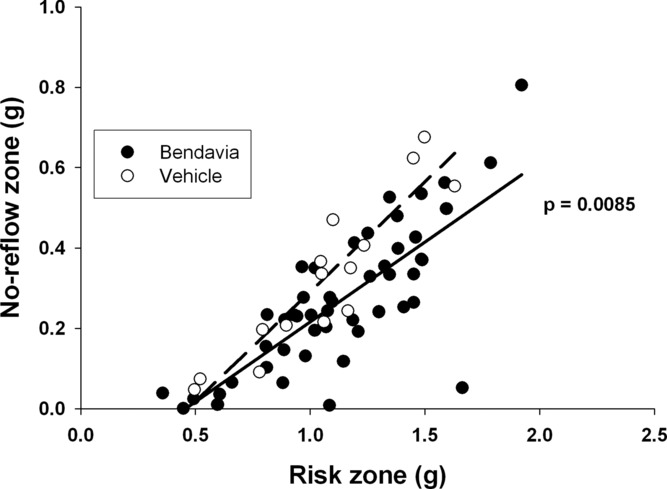
Relationship between risk zone (g) and the extent of no-reflow (g). Lines of regression for vehicle-treated rabbits (dashed line) and Bendavia-treated rabbits (solid line) are shown. *P=0.0085 by ANCOVA testing for a treatment effect (Bendavia vs vehicle) on the relationship.

In the rabbit model, Bendavia significantly reduced the extent of no-reflow expressed as a percentage of the risk zone when the relationship between the extent of the risk zone and the extent of the no-reflow area in the 4 groups was analyzed by ANCOVA testing for group as a covariate effect (*P*=0.036). The mean value of no-reflow, expressed as a fraction of the risk region, was 0.22±0.02 in the combined Bendavia groups versus 0.28±0.03 in the control group (*P*=0.07). There was a significant group effect on the relationship between the no-reflow zone and the risk zone (*P*=0.0085, combined Bendavia versus vehicle; Figure 4). The regression line for the treated hearts lies below that of the control hearts. That is, for any given risk zone, the extent of no-reflow was significantly smaller in Bendavia-treated animals.

### Effects of Bendavia on Postischemia Hemodynamic Recovery and Arrhythmia

Across models, Bendavia had no major effects on the recovery of cardiac electromechanical function after ischemia/reperfusion. In sheep studies, treatment with Bendavia had no significant effect on heart rate, mean arterial pressure, mean pulmonary artery pressure, pulmonary wedge pressure, central venous pressure, or cardiac output as compared with the vehicle group. In rabbit studies, there were no significant differences among groups in heart rate or blood pressure.

In guinea pig experiments, neither the incidence of arrhythmia nor the extent of recovery of LV function was influenced by Bendavia treatment. Arrhythmia scores for the 2-hour reperfusion period were 4.9±0.4 in the control group (n=14). Administration of Bendavia, either before ischemia or at the onset of reperfusion, had no effect on arrhythmia scores (5.8±0.7 and 4.4±0.4, respectively, n=9 and 9, respectively; *P*>0.05, ANOVA). Cyclosporin A treatment also had no significant effect on the incidence of arrhythmia (arrhythmia score of 4.0±0.5, n=8; *P*>0.05, ANOVA). LV developed pressure at the end of the 2-hour protocol was 23±5 mm Hg in the control group (n=14) and 24±6 mm Hg when Bendavia was administered during both ischemia and reperfusion (n=9). Administration of Bendavia or cyclosporine A at the onset of reperfusion also had no effect on recovery of developed pressure (39±5 and 34±6 mm Hg, n=9 and 8, respectively; *P*>0.05, ANOVA).

### Myocardial Uptake of Bendavia

**Figure 5.**
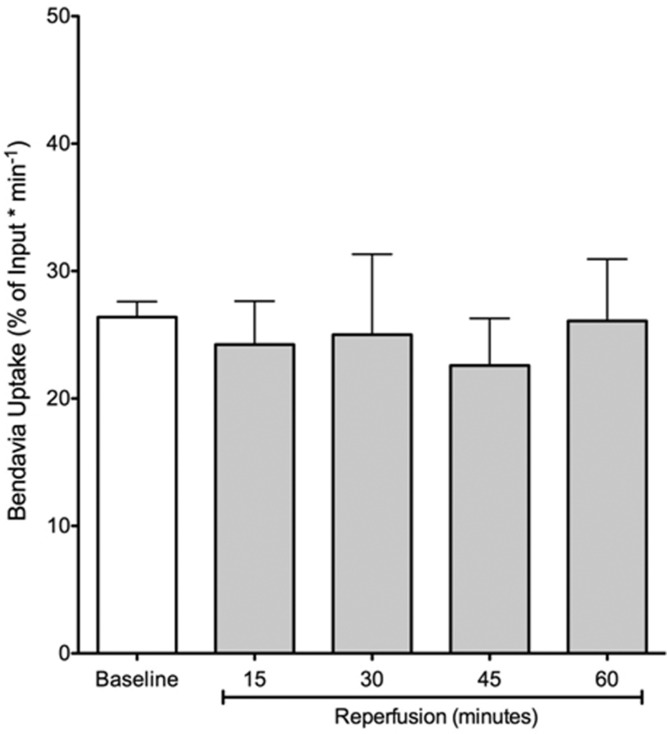
Uptake of Bendavia by the myocardium before ischemia (Baseline) and during reperfusion. Data represent the percentage of Bendavia taken up by the heart over a 1-minute time course.

Cardiac uptake of Bendavia is presented in Figure 5. The guinea pig myocardium extracted ≈25% of Bendavia per minute from the perfusate, and there were no differences in the rate of uptake between baseline perfusion and the reperfusion period (*P*>0.05, ANOVA).

### Guinea Pig Myocyte Survival During Hypoxia/Reoxygenation

**Figure 6.**
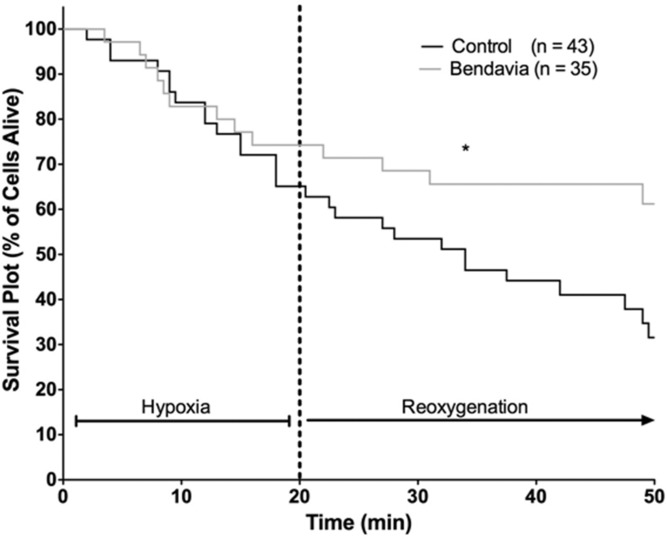
Survival plot for myocytes in the study exposed to hypoxia and reoxygenation. Each cell death event is noted as a downward step in the survival curve. *P<0.02, log-rank (Mantel-Cox) test vs control for the reoxygenation period. Bendavia significantly lowered cell death rate during reoxygenation, but the extent of cell death during hypoxia was similar to control.

In this study, a total of 78 guinea pig cardiac myocytes were exposed to cellular hypoxia/reoxygenation. Because cells died during both the hypoxic and reoxygenation periods, a cell survival plot is presented in Figure 6. A total of 43 cells were exposed to hypoxia/reoxygenation under control (no-drug) conditions, whereas another 35 cells were treated with 1 nmol/L Bendavia. At the end of the 20-minute hypoxia period, 65% of control cells (28 of 43 total) and 74% of Bendavia-treated cells (26 of 35 total) were still alive (no drug effect during hypoxia; *P*>0.05, Mantel-Cox test). Bendavia specifically prevented death associated with reoxygenation. After reoxygenation, there was a significant decrease in cell death evoked by Bendavia. Of the cells that were alive at the onset of reoxygenation, only 54% of control cells survived until the end of reoxygenation, compared to 85% of cells that were treated with Bendavia (*P*<0.02, Mantel-Cox test).

### Role of Mitochondrial ROS in Cell Death

**Figure 7.**
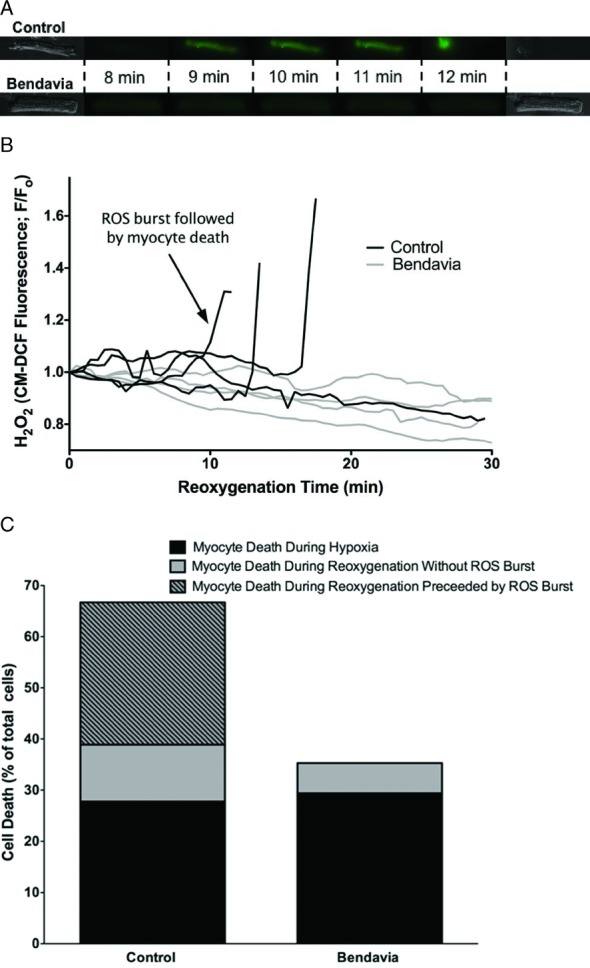
Cellular ROS production during hypoxia/reoxygenation. A, Representative fluorescence images of cardiac ventricular myocytes loaded with the ROS sensor CM-DCF. ROS bursts during reoxygenation preceded cell death, and Bendavia treatment prevented ROS-induced cell death. B, Representative fluorescence intensity traces for cells in the study. CM-DCF fluorescence is normalized to the basal fluorescence (F_o_) for each cell at the end of hypoxia. C, Contribution of ROS bursts to myocyte death during hypoxia/reoxygenation.

A subset of myocytes (n=35 total) exposed to hypoxia/reoxygenation were loaded with the ROS sensor CM-DCF during the protocol to monitor ROS production with (n=18) or without (n=17) Bendavia pretreatment. Figure 7 depicts the incidence of a preceding ROS burst before cell death. The majority of cell death in the control group was preceded by a burst of ROS during the reoxygenation period. ROS-dependent cell death was completely prevented in myocytes treated with Bendavia (Figure 7B and 7C). ROS-independent cell death during hypoxia or reoxygenation was similar between the control and Bendavia-treated groups. The time course for ROS bursts in our model (as assessed by DCF fluorescence) ranged between 1 and 19 minutes of reperfusion, with most bursts occurring between minutes 5 and 15 of reoxygenation.

### Maintenance of Mitochondrial Membrane Potential (ΔΨ_m_)

**Figure 8.**
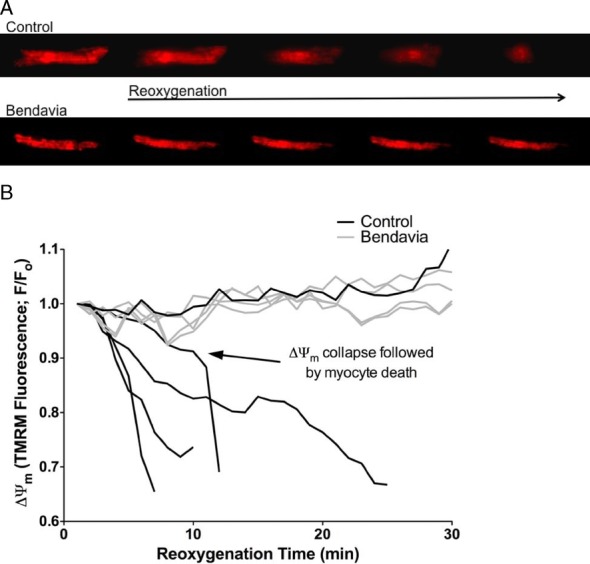
Mitochondrial membrane potential (ΔΨ_m_) in myocytes during cellular hypoxia/reoxygenation. A, Representative fluorescence images of myocytes loaded with the ΔΨ_m_ sensor TMRM. ΔΨ_m_ collapse often preceded cell death, and treatment with Bendavia improved the capacity to maintain ΔΨ_m_. B, Representative fluorescence intensity traces for cells in the study.

Another subset of myocytes (n=37 total) was exposed to hypoxia/reoxygenation while loaded with the ΔΨ_m_ indicator TMRM (presented in Figure 8). Myocytes treated with Bendavia were protected against cell death during reoxygenation, and specifically against cell death that was preceded by a collapse of ΔΨ_m_. Only 14% of cells in the Bendavia-treated group displayed a collapse in ΔΨ_m_ before cell death, compared to 42% of untreated cells. These data suggest that Bendavia helped promote cell survival by sustaining mitochondrial energetics (ΔΨ_m_) during reoxygenation and decreasing the overall incidence of permeability transition.

## Discussion

### Key Findings

Numerous agents have been tested as adjunctive therapy for reperfusion injury in the setting of acute myocardial infarction. At the present time, early reperfusion via thrombolytic therapy or percutaneous coronary intervention (including percutaneous transluminal coronary angioplasty) is the only accepted definitive therapy for acute myocardial infarction.^22^ Early reperfusion limits myocardial infarct size and improves survival rate. Nevertheless, not all patients receive early coronary artery reperfusion, and at the time of reperfusion some degree of injury may occur as a result of reperfusion itself. Stunned myocardium, the no-reflow phenomenon, microvascular injury, and reperfusion arrhythmias are forms of reperfusion injury.

The present studies show that Bendavia, administered *after* the onset of ischemia, demonstrated cardioprotective effects in several diverse models of ischemia/reperfusion. Bendavia reduced myocardial infarct size in a large animal model (sheep), attenuated the extent of no-reflow in an in vivo rabbit model, and reduced infarct size in isolated guinea pig hearts. Bursts of ROS were blunted by Bendavia, resulting in better maintenance of mitochondrial energetics and lowered cell death during reoxygenation. Finally, we observed that myocardial uptake of Bendavia is high even during early reperfusion, indicating that this compound is actively taken up by myocardium even if the tissue has been exposed to an ischemic insult.

### Cardioprotection at Reperfusion: “Salvaging Cells That Are Salvageable”

Cell death during ischemia and reperfusion is multifactorial and is generally attributed to a combination of necrosis and apoptosis,^23^ with necrotic cell death being the predominant cause of death in ischemic myocardium.^24^ Although reperfusion is requisite to salvage tissue, prompt reperfusion also may injure cells that are hovering between life and death. Elevated intracellular levels of calcium, sodium, and inorganic phosphate, an alkaline shift toward physiological pH, and production of ROS are all noted in early reperfusion.^23^ These factors promote the opening of energy-dissipating channels in the inner mitochondrial membrane. In particular, the open probability of both the mitochondrial permeability transition pore (reviewed in Halestrap et al^10^) and the inner membrane anion channel (reviewed in Brown et al 2010^25^) is greatly enhanced by ROS. Oxidant-induced pore/channel opening collapses mitochondrial membrane potential (ΔΨ_m_), leading to cessation of adenosine triphosphate production and ultimately cell death.

### The Mitochondria as a Therapeutic Target

In the clinical realm, heightened ROS production is implicated in the development of both infarction and the microvascular damage that underlies coronary no-reflow.^8,23^ There is obviously a great deal of interest in decreasing reperfusion injury with compounds that scavenge reactive intermediates or directly block the permeability transition pore. Among the candidate radical scavengers tested to date, early efforts focused primarily on superoxide dismutase mimetics and catalase. These approaches reduced infarct size in some^26–29^ (but not all^30^) animal studies and also decreased microvascular damage and reduced no-reflow.^30^ Despite promising results in animal studies, these strategies do not appear to translate to beneficial effects in clinical trials.^31,32^ The reasons for the lack of translation to the clinic have been described in detail elsewhere^20,33^ but likely involve cell permeability concerns, as well as findings that nonspecific radical scavengers must be used in very high doses for efficacy to be seen. Furthermore, superoxide dismutase mimetics scavenge only superoxide anion, whereas a significant portion of tissue injury may arise from radical-independent redox signaling (ie, oxidative shifts in intracellular thiols).^34^

Direct permeability transition pore blockers have recently shown potential in reducing reperfusion injury in animal and human studies. Cyclosporin A, which inhibits the association of cyclophilin D with the mitochondrial permeability transition pore, has been shown to reduce cardiac ischemia/reperfusion injury^35,36^ (Figure 3). In a recent small clinical trial,^21^ cyclosporine given at the time of percutaneous coronary intervention significantly reduced infarct size (assessed with both magnetic resonance imaging and enzymatic markers in serum) in humans, corroborating previous data from studies in animals. A multicenter clinical trial is currently under way in Europe to investigate cyclosporin in a larger population of myocardial infarct patients. Although these early results are promising, the use of cyclosporin may be limited by a narrow therapeutic window,^37^ nonspecific effects on other cellular cyclophilins/calcineurin,^38^ and reports of cyclosporin-induced vasoconstriction.^39^

### Reduction of Infarction With Bendavia

A clear need exists for cytoprotective compounds that freely cross the sarcolemma, are effective across low doses, and specifically target the region within the cell where oxidant production is high (specifically, the mitochondria). Drs Hazel H. Szeto and Peter W. Schiller developed a unique class of peptides that concentrate within mitochondria and reduce ROS generation in cultured cells exposed to chemical oxidative stress.^13,40^ Bendavia (an analogue of SS-02 and SS-31 in the literature) is a novel peptide that is able to cross cell membranes. In particular, studies using colocalization confocal imaging and radiolabeled peptide uptake indicated that Bendavia localizes to (and accumulates within) mitochondria,^12,41^ making it an attractive therapy to reduce mitochondria-derived ROS.

From a therapeutic standpoint, Bendavia uptake appears to be independent of the ΔΨ_m_. ΔΨ_m_ collapses during ischemia, and the recovery of ΔΨ_m_ at reperfusion is very heterogeneous in the myocardium.^42,43^ Treatment strategies that require ΔΨ_m_ for mitochondrial delivery (such as antioxidants conjugated to triphenylphosphonium cations, eg, SkQ1, MitoQ, MitoE, MitoSOD^44^) may only be targeting only cells that are already on their way to recovery. In our study, myocardial uptake of Bendavia was observed even in early reperfusion (when ΔΨ_m_ may be compromised), consistent with studies in isolated cells where accumulation was not markedly affected by chemical uncouplers of mitochondria.^12^ Furthermore, the ΔΨ_m_-depolarizing effects of compounds tethered to triphenylphosphonium make them self-limiting and translate to a very narrow therapeutic window of efficacy.^13^ Bendavia treatment has no noticeable effect on basal ΔΨ_m_ when assessed by either TMRM fluorescence^12^ or triphenylphosphonium uptake (D.A. Brown, unpublished observations), allowing for a very wide therapeutic range and cardioprotection at or below nanomolar concentrations (Zhao et al 2004^12^ and herein).

In the present study, we show for the first time that Bendavia treatment reduced cellular ROS generation and helped sustain ΔΨ_m_ in primary cardiac myocytes exposed to hypoxia/reoxygenation. In particular, Bendavia reduced oxidant-dependent cell death during the reoxygenation period yet had no effect on myocyte survival during hypoxia. This finding supports our hypothesis that this compound is most effective when production of ROS is high and needs to be suppressed or ablated at the site of generation (ie, the mitochondria). The time course of ROS burst and myocyte death that we observed is generally consistent with studies in the literature that have used both in vivo and isolated heart models. Two different studies found that the burst of ROS (assessed by electron spin resonance spectroscopy) remained high for the first 20 minutes of reperfusion, after which the burst waned.^45,46^ In both of these studies, ROS levels peaked within the first 5 minutes and remained significantly elevated through the first 20 minutes of reperfusion. We also found that ROS bursts were highest in the first 20 minutes of reoxygenation, although the timing of ROS bursts was slightly delayed in our model compared to intact systems (most ROS-dependent cell death occurred between minutes 5 and 15 of reoxygenation in our study). A likely explanation for this discrepancy is that our isolated myocytes were not loaded, and thus the energetic needs of the myocytes were lower than they would be in an intact syncytium, which ostensibly prolonged the tolerance to metabolic insult in our model.

Our myocyte data corroborate previous work in cell culture models, where Bendavia lowered the levels of ROS and promoted cellular survival in neuronal cells exposed to t-butylhydroperoxide.^12^ It seems plausible that by keeping ROS low, either by inhibiting the initiation and cascade of ROS production or by mitochondrial ROS scavenging, Bendavia maintains ΔΨ_m_ by reducing the open probability of (1) energy-dissipating ion channels in the inner membrane and (2) the permeability transition pore. With regard to the effects of Bendavia on the permeability transition pore, we recently directly examined the effects of Bendavia on calcium-induced permeability transition pore opening in isolated mitochondria. Bendavia does not appear to be a direct blocker of the pore, as opposed to blockers such as cyclosporin-A or NIM811.^47^ Therefore, the maintenance of myocyte energetics (ΔΨ_m_) that we observed in our study seems to be an indirect effect of Bendavia (by keeping ROS low), rather than a direct effect on the pore.

The cardioprotection that we observed in isolated guinea pig cells translated to infarct-size reduction in several models of ischemia/reperfusion. Notably, in each case, the protection was observed when Bendavia was given after the onset of ischemia. These data support the concept that damage to the mitochondria at the time of reperfusion is therapeutically preventable.

### Bendavia Protection Against the No-Reflow Phenomenon

Our study is also the first to demonstrate a beneficial effect of Bendavia on the no-reflow phenomenon. No-reflow is the phenomenon whereby portions of a myocardial infarction cannot be reperfused despite the fact that the epicardial infarct-related artery has been successfully reopened and remains patent. The cause of no-reflow is primarily microvascular obstruction due to localized and regional swelling of the endothelium. No-reflow has been identified in humans after myocardial infarction by using a number of techniques, including magnetic resonance imaging, echo contrast, and nuclear scanning.^48^ Because the zone of no-reflow is cut off from blood supply, no-reflow may impede the healing phase of infarction. In both experimental and clinical studies, no-reflow is associated with more myocardial infarct expansion, worse LV dilation and remodeling, and more congestive heart failure and death.^5,48^ At the present time, there are no FDA-approved drugs for the treatment of no-reflow. In our study, for any given ischemic risk zone size, Bendavia decreased no-reflow. This independent effect on no-reflow for any given risk zone is one of the first demonstrations of its kind for a cardioprotective agent in the present model and is consistent with results previously seen only with hypothermia induced before reperfusion.

Why should an agent that targets the mitochondria have this effect on the vasculature? Mitochondrial ROS production by endothelial cells during early reperfusion contributes to microvascular damage associated with no-reflow.^49^ Isolated endothelial cells exposed to hypoxia/reoxygenation display increased mitochondrial ROS levels after reoxygenation, and inhibitors of mitochondrial electron flow reduced endothelial ROS production.^50^ Our study is the first to extrapolate these findings from cultured cells to an intact mammalian (rabbit) model of ischemia/reperfusion, demonstrating that targeting and preventing endothelial mitochondrial ROS during early reperfusion functionally reduces no-reflow. Moreover, our use of Bendavia, which is effective at low doses and has no effect on normal mitochondrial respiration,^12^ represents a significant advancement over more toxic approaches that reduce endothelial ROS by directly blocking mitochondrial respiration.

### Study Limitations

The reduction of myocardial infarct size in the sheep model was modest, as was the trend toward reduction in the rabbit model. In the isolated guinea pig hearts, the reduction in infarct size was more robust. Although other drugs may have shown greater reductions in infarct size,^51,52^ the directional change was similar in 3 separate species studied in 3 separate laboratories. It is possible that small sample sizes might explain the fact that some of the comparisons failed to reach statistical significance. Whether Bendavia would have any effect in patients is unknown.

There are examples in the clinical literature in which the reduction of infarct size was modest—for example, remote ischemic conditioning, which reduced troponin T release by 16%.^53^ Several meta-analyses have examined the reduction in infarct size associated with bone marrow–derived stem cell therapy, showing modest reductions of infarct size of 6% and 7%,^54,55^ but this was associated with long-term improvement in ejection fraction. We would not expect improvement in functional recovery in the type of short-term studies described in the present article because the salvaged peri-infarct myocardium remains stunned for days to weeks after reperfusion. Hence, to determine whether Bendavia improves cardiac function in an infarct model would require a long-term study. We did not specifically study Bendavia in a stunned myocardium model, which would require studying a brief period of transient ischemia (5 to 15 minutes) that is not associated with necrosis. Another critical component of ischemia/reperfusion injury is related to calcium overload, which contributes to opening of the mitochondrial permeability transition pore.^56^ We did not specifically study whether Bendavia limits calcium overload, but this would be of interest in future studies.

### Summary

In conclusion, the mitochondria-targeting agent Bendavia demonstrated cardioprotective properties in several in vitro and in vivo experimental models when administered before reperfusion. It protected cardiomyocytes; it limited myocardial infarct size; and for the first time it was shown to limit no-reflow.
